# Population Pharmacokinetics of Oxycodone and Metabolites in Patients with Cancer-Related Pain

**DOI:** 10.3390/cancers13112768

**Published:** 2021-06-02

**Authors:** Bram C. Agema, Astrid W. Oosten, Sebastiaan D.T. Sassen, Wim J.R. Rietdijk, Carin C.D. van der Rijt, Birgit C.P. Koch, Ron H.J. Mathijssen, Stijn L.W. Koolen

**Affiliations:** 1 Department of Medical Oncology, Erasmus MC Cancer Institute, Erasmus University Medical Center, dr. Molewaterplein 40, 3015GD Rotterdam, The Netherlands; a.oosten@erasmusmc.nl (A.W.O.); c.vanderrijt@erasmusmc.nl (C.C.D.v.d.R.); a.mathijssen@erasmusmc.nl (R.H.J.M.); s.koolen@erasmusmc.nl (S.L.W.K.); 2 Department of Clinical Pharmacy, Erasmus University Medical Center, dr. Molewaterplein 40, 3015GD Rotterdam, The Netherlands; s.sassen@erasmusmc.nl (S.D.T.S.); w.rietdijk@erasmusmc.nl (W.J.R.R.); B.koch@erasmusmc.nl (B.C.P.K.)

**Keywords:** oxycodone, opioids, pharmacokinetics, noroxycodone, noroxymorphone, modelling, cancer-related pain

## Abstract

**Simple Summary:**

Patients with moderate to severe cancer-related pain are frequently treated with oxycodone, a strong-acting opioid. However, treatment with oxycodone does not always lead to sufficient analgesic action. In order to determine which factors affect treatment outcomes, we performed an observational study and developed a population pharmacokinetic model. The model described oxycodone, nor-oxycodone and nor-oxymorphone pharmacokinetics. The association between oxycodone or oxycodone metabolites’ exposure with pain scores and adverse events was not significant. The combined oxycodone, nor-oxycodone and nor-oxymorphone model is a good starting point for further unravelling the factors that affect the pharmacokinetic/pharmacodynamic relation of oxycodone and its metabolites.

**Abstract:**

Oxycodone is frequently used for treating cancer-related pain, while not much is known about the factors that influence treatment outcomes in these patients. We aim to unravel these factors by developing a population-pharmacokinetic model to assess the pharmacokinetics of oxycodone and its metabolites in cancer patients, and to associate this with pain scores, and adverse events. Hospitalized patients with cancer-related pain, who were treated with oral oxycodone, could participate. Pharmacokinetic samples and patient-reported pain scores and occurrence and severity of nine adverse events were taken every 12 h. In 28 patients, 302 pharmacokinetic samples were collected. A one-compartment model for oxycodone and each metabolite best described oxycodone, nor-oxycodone, and nor-oxymorphone pharmacokinetics. Furthermore, oxycodone exposure was not associated with average and maximal pain scores, and oxycodone, nor-oxycodone, and nor-oxymorphone exposure were not associated with adverse events (all *p* > 0.05). This is the first model to describe the pharmacokinetics of oxycodone including the metabolites nor-oxycodone and nor-oxymorphone in hospitalized patients with cancer pain. Additional research, including more patients and a more timely collection of pharmacodynamic data, is needed to further elucidate oxycodone (metabolite) pharmacokinetic/pharmacodynamic relationships. This model is an important starting point for further studies to optimize oxycodone dosing regiments in patients with cancer-related pain.

## 1. Introduction

Pain is the most common symptom in cancer patients and its incidence increases with disease progression. Pain prevalence rates were 39.3% after curative treatment, 55% during anticancer treatment and 66% in advanced, metastatic, or terminal disease [1]. To treat severe to moderate cancer-related pain, the World Health Organization recommends strong opioids [2]. 

Oxycodone is a widely used strong-acting opioid-agonist and is available in immediate-release (IR) and extended-release (ER) formulations. This combination is practical in treating both chronic and breakthrough pain with the same drug. However, adequate pain-control is not always achieved. Pain relief and the occurrence of adverse events vary largely between patients [1,2]. Because of the variation in both pain relief and the occurrence of adverse events, clinical practice mostly consists of dose-titration until either sufficient analgesic effect is achieved or dose-limiting adverse events occur [3]. In case of insufficient pain control and/or dose-limiting adverse events, opioid rotation is indicated. This process is conducted on an empirical basis for each patient and is often time consuming. 

The variation in analgesic effect and adverse events could, in part, be explained by differences in pathogenesis and therefore differences in pathophysiology and sensation of pain. Moreover, inter- and intra-individual variation in oxycodone exposure could contribute to this variation in pain relief and the occurrence of adverse events. Factors such as age, sex, organ functions, and genetic polymorphisms have been shown to cause differences in oxycodone exposure [4,5,6]. However, these findings are mostly inconsistent among scientific reports. 

**Figure 1:**
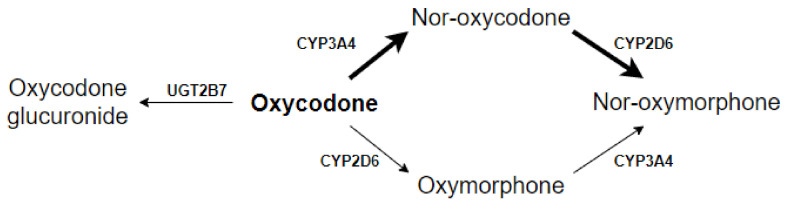
Schematic representation of oxycodone metabolism [6].

Oxycodone metabolites could also contribute to the analgesic effect and the occurrence of adverse events and cause this variation. However, the exact contribution of oxycodone metabolites to the analgesic action of oxycodone is unclear. Oxycodone is mainly metabolized by CYP2D6 to oxymorphone and by CYP3A4 to nor-oxycodone. Another part is excreted after glucuronidation by UGT2B7. Both oxymorphone and nor-oxycodone are subsequently metabolized into nor-oxymorphone (Figure 1) [4,7]. All metabolites have an affinity for the μ-opioid receptor; however, the exact contribution of each metabolite to the analgesic effect is unknown [8]. The affinity of oxymorphone to μ-opioid receptors is 10- to 45-fold higher than oxycodone, and oxymorphone is therefore believed to contribute to the nociceptive effect despite low concentrations in both the plasma as the cerebrospinal fluid [9]. Both nor-oxycodone and nor-oxymorphone poorly cross the blood–brain barrier and are therefore not likely to contribute to the analgesic effect of oxycodone. However, because of their relatively high plasma concentrations and affinity for the μ-opioid receptor, the oxycodone metabolites could, theoretically, also contribute to the variation in the occurrence of adverse events by stimulating peripheral μ-opioid receptors [10]. 

Earlier pharmacokinetic/pharmacodynamic (PK/PD) models have been developed to explain and quantify the variation in the treatment outcomes. However, these models were mostly developed using data of oxycodone concentrations in healthy volunteers and patients, without comorbidities, who had undergone surgery [5,11,12,13,14,15,16]. One study has developed a population pharmacokinetic model of oxycodone in patients with cancer-related pain. However, no metabolites were incorporated in the model and no pharmacodynamic data were collected [17]. To date, no population PK/PD study has been conducted to assess the pharmacokinetics and pharmacodynamics of oxycodone and its metabolites in cancer patients. 

Taken together, our aim is to develop a pharmacokinetic model for oxycodone and its metabolites including the effect of covariates in a sample of hospitalized cancer patients. Further, as a secondary aim, we associate oxycodone and metabolite exposure with clinical endpoints, i.e., pain and adverse events in this population. This study may contribute to unraveling factors and be a starting point for the precision dosing of oxycodone in cancer patients.

## 2. Materials and Methods

### 2.1. Study Design and Study Population

All patients who were treated with oxycodone for moderate to severe nociceptive cancer-related pain at the Erasmus Medical Center Cancer institute were asked to participate in this observational study. All patients treated with oxycodone were eligible, i.e., patients already treated with oxycodone before admission but also opioid-naïve patients or patients rotating to oxycodone after failure of treatment with another opioid. Patients treated with a fentanyl patch who were prescribed IR oxycodone could also be included in the study. Patients were included as soon as possible after hospital admission or at the start of opioid treatment during hospitalization. The study was approved by the Erasmus Medical Center Medical Ethics Review Committee (MEC 09.332). Written informed consent was obtained from all participants. The trial was registered in the Dutch Trial Registry (registration ID: NTR4369).

At inclusion, baseline characteristics were collected. The baseline characteristics are age, sex, weight, height, cancer type, AST, ALT, bilirubin, albumin, glomerular filtration rate, CYP2D6, CYP3A4*22, and UGT2B7 genotypes and comorbidities. As we performed an observational study, oxycodone starting doses at admission were based on, if present, previous treatments and otherwise on clinical assessment of the treating physician. The intensity of both maximal and average pain in the last 12 h was measured twice daily on the 0–10 Numeric Rating Scale Additionally, nine common side effects were assessed twice daily using a 4-point Likert scale [18]. These side effects were nausea, vomiting, dry mouth, drowsiness, obstipation, myoclonia, confusion, hallucinations and transpiration. Co-medication of all patients was screened for moderate and strong CYP3A4 and CYP2D6 inhibitors and inducers and drug–drug interactions affecting oxycodone pharmacokinetics (Table S1). Patients completed the study when they were discharged from the hospital or when stable pain control was reached during 72 h.

### 2.2. Pharmacokinetic Sample Collection

After inclusion, plasma samples were taken twice daily at 8 AM and 8 PM. The oxycodone ER tablets were also administered at these times. Maximally once a day, samples before and 5, 15, 30 and 60 min after administration of an oxycodone IR tablet were taken to identify the absorption phase of the IR tablets. Plasma samples were collected in potassium EDTA tubes. After centrifugation, the supernatant was collected and stored at −70 °C until analysis at the laboratory of Translational Pharmacology (Erasmus MC Cancer Institute). 

### 2.3. Determination of Oxycodone and Metabolites

Plasma concentrations of oxycodone and its metabolites, oxymorphone, nor-oxycodone and nor-oxymorphone were quantified using a validated UPLC-MS/MS method consisting of a Waters Acquity UPLC sample manager coupled to a triple quadrupole mass spectrometer operating in the multiple reaction monitoring mode (MRM) with positive ion electrospray ionization (Waters, Etten-Leur, The Netherlands). Chromatographic separations were achieved on an Acquity UPLC^®^ (Waters, Etten-Leur, The Netherlands) BEH C18 1.7 µm 2.1 × 100 mm column eluted at a flow rate of 0.350 mL/min on a gradient of methanol combined with ammonium formate. The overall cycle time was 10 min. The calibration curves were linear with the lower limit of quantitation validated at 0.200 ng/mL for oxycodone and oxymorphone and 1.00 ng/mL for nor-oxycodone and nor-oxymorphone. 

### 2.4. Population Pharmacokinetic Analysis

The pharmacokinetic data were analyzed using non-linear mixed-effects modelling (NONMEM) (version 7.4, ICON, Development Solutions, Ellicott City, MD, USA). The Laplacian Estimation with Interaction method was used. During model development and evaluation, we also used Perl-speaks-NONMEM version 4.2.0 [19,20], Pirana software version 2.9.5b (Certara, NJ, USA) [21], R version 4.0.2 (R Foundation for Statistical Computing, Vienna, Austria) and, Xpose version 4.4.1 [22].

At first, a one-compartment model with first-order absorption for oxycodone was constructed. Several model components were tested, including different mechanistic absorption models, such as transit compartment models, multiphasic absorption, lag time, but also different estimations of plasma concentrations at the start of the study, when patients also used oxycodone prior to hospital admission. 

The residual error was estimated using a combined proportional and additive error model. Inter-individual variability of pharmacokinetic parameters was modelled using exponential models. The M3 method was used if more than 10% of the samples from a single compound were below the limit of quantification (BLQ) [23,24]. This method censors the BLQ data and introduces a likelihood that the plasma concentrations at that time point are indeed BLQ.

Continuous covariates were centered on the median and were modeled as exponential and power models. Categorical covariates were modeled as proportional models, while genotypes were modeled as exponential, dominant, and recessive models (Document S1) [25]. Covariate analysis was performed using stepwise forward inclusion (*p* < 0.05) and backwards elimination (*p* < 0.01).

### 2.5. Model Evaluation

The model was evaluated numerically by changes in the objective function value (OFV) and a nonparametric bootstrap procedure (*n* = 1,000). Changes that result in an OFV decrease greater than 3.84 for one degree of freedom were considered significant (*p* < 0.05). Models were evaluated visually using goodness-of-fit (GOF) plots, visual predictive checks (VPC) plots and normalized prediction distribution errors (NPDE) plots [26]. In addition, the 95% confidence interval of the estimation of the covariate should not include 0 to be deemed significant. 

### 2.6. Pharmacodynamic Analysis

For pharmacodynamic modelling, the linear, Emax, direct effect, effect-compartment and turnover models as previously described by Upton et al. were used to test exposur–effect relationships [27]. Patients that used other opioids concomitantly with oxycodone were excluded in the pharmacodynamic analysis. Additionally, as patients rated the average and maximal pain in the last 12 h, the pain scores collected at inclusion in the study were not included in this analysis.

In addition to the pharmacodynamic model, associations between drug exposure, quantified as area under the curve (AUC) in the 12 h prior to reporting of the pain-scores and adverse events, were tested using mixed-effects linear regressions. We used the pain scores and the sum of adverse event when scored as outcome. The adverse events were scored from 0 (not present) to 3 (severe). Both pain scores and the sum of all adverse events were used as outcomes. The mixed-effects linear regressions were performed in R version 4.0.2 with lme4 [28], where beta’s and the 95% confidence interval were estimated.

## 3. Results

### 3.1. Patients 

In total, 302 pharmacokinetic samples for oxycodone and its metabolites were available for analysis from 28 patients. One patient participated in the study twice. All four compounds were quantified in these samples which resulted in 1207 separate measurements. In total, 201 patient-reported pain scores (both maximum and average pain) were collected and adverse events were registered 185 times. Two of the patients used a moderate CYP3A4 inhibitor (imatinib and diltiazem) at the start of the study. Excluding these patients did not alter the model significantly and were therefore included in the study. None of the patients was a poor metabolizer or ultrarapid metabolizer for CYP2D6 or CYP3A4 homozygous variant. None of the patients showed liver dysfunction classified as Child-Pugh classification B or C. In three patients, the estimated glomerular filtration rate (eGFR) was less than 60 mL/min. See Table 1 for all patient characteristics. Doses varied between 5 and 100 mg twice daily for extended-release tablets and between 5 and 30 mg for immediate-release tablets. 

In total, 352 out of the 1207 measurements were BLQ (29.2%). Only 6.5% of all measurements were censored. Sixty-nine measurements were censored because of a (single) missing oxycodone administration, and all ten nor-oxycodone measurements, from a single patient, were discarded after structural co-administration with dexamethasone; a weak CYP3A4 inducer [29]. At the same time as the start of dexamethasone co-administration, the oxycodone dosage was raised from 60 to 100 mg oxycodone twice daily. Oxycodone, oxymorphone and nor-oxymorphone plasma concentrations increased as expected. However, nor-oxycodone plasma concentrations decreased structurally from approximately 100 to 15 nM. Other patients that used dexamethasone used it throughout the study and a comparable effect was not observed. Including the nor-oxycodone samples, of the patient that used dexamethasone concomitantly, altered the parameter precision in the final model significantly. These samples were therefore excluded.

### 3.2. Population Pharmacokinetic Analysis

**Figure 2:**
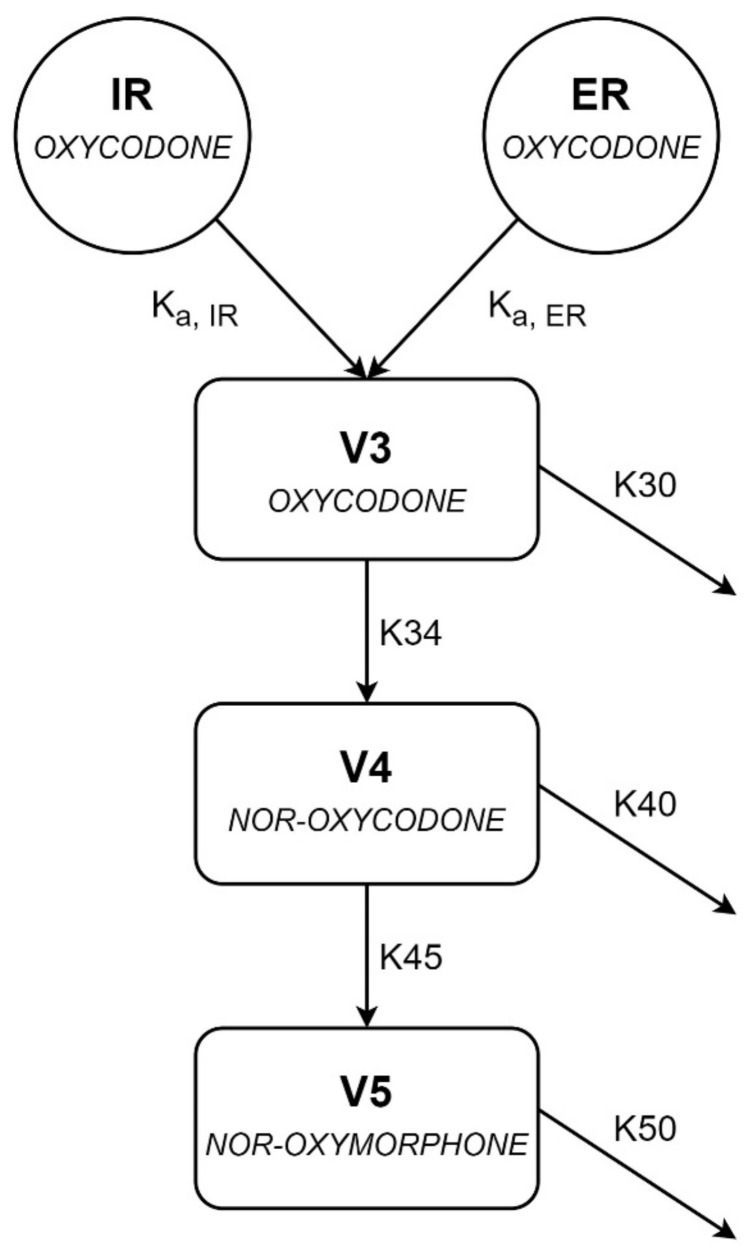
Schematic representation of the joint population pharmacokinetic model of oxycodone, nor-oxycodone and nor-oxymorphone. ER and IR are the compartments for the extended-release and the immediate-release oxycodone tablets. Rate constants K_A,IR_ and K_A,ER_ are the absorption rates for IR and ER tablets. K30, K40 and K50 are the elimination rate constants for oxycodone, nor-oxycodone, and nor-oxymorphone, and V3, V4 and V5 are the distribution volumes for oxycodone, nor-oxycodone, and nor-oxymorphone.

A one-compartment model for oxycodone nor-oxycodone and nor-oxymorphone, with first-order absorption of IR and ER oxycodone, first-order formation of metabolites and first-order elimination, was developed (Figure 2 and Document S2). Inter-individual variability was included on the conversion rate from oxycodone to nor-oxycodone, nor-oxycodone clearance and nor-oxymorphone clearance. All parameter estimates are shown in Table 2.

Oxycodone absorption was best described using two separate dosing compartments for IR and ER oxycodone administrations. The data were adequately described by first-order absorption rates. Modelling lag times, biphasic absorption for ER tablets, estimation of plasma concentration at inclusion and different bio-availabilities for IR and ER oxycodone tablets did not improve the model significantly. Two patients already had detectable oxycodone concentrations at inclusion. As no information about oxycodone administrations prior to the hospital admission was available, these pharmacokinetic samples were included in the model as starting concentrations. We also tried to use steady-state concentrations as starting concentrations using the new dosing regimen. This, however, resulted in significantly lesser model stability.

The elimination rate constant (K30) for oxycodone and the distribution volumes of nor-oxycodone and nor-oxymorphone could not be adequately estimated and were therefore fixed based on intermediate models. Because this is the first compartmental model to describe nor-oxycodone and nor-oxymorphone, no values were present in literature to fix the parameters upon. Fixing these parameters on intermediate models minimally compromised the fit of the model, which was evaluated by goodness-of-fit plots.

Oxymorphone could not be incorporated into the model because 179 out of 309 oxymorphone measurements were BLQ. For twelve patients, the oxymorphone concentrations were BLQ in all samples. When modelled, oxymorphone parameters showed high shrinkage (>20%), and poor parameter precision (residual standard error (RSE) > 100%). In order to account for nor-oxymorphone formation out of oxymorphone (Figure 1), an extra clearance from oxycodone to nor-oxymorphone was modeled. This, however, resulted in a 1.4 × 10^11^ increase in conditional number and was not incorporated in the final model. 

The residual error for nor-oxycodone and nor-oxymorphone was best described by a combined additive and proportional error model for each compound. Because the additive error model introduced instability for oxycodone estimations, the additive error was fixed to 0 (Table 2). 

None of the covariates significantly improved the model when included and were therefore not incorporated into the model. 

**Figure 3:**
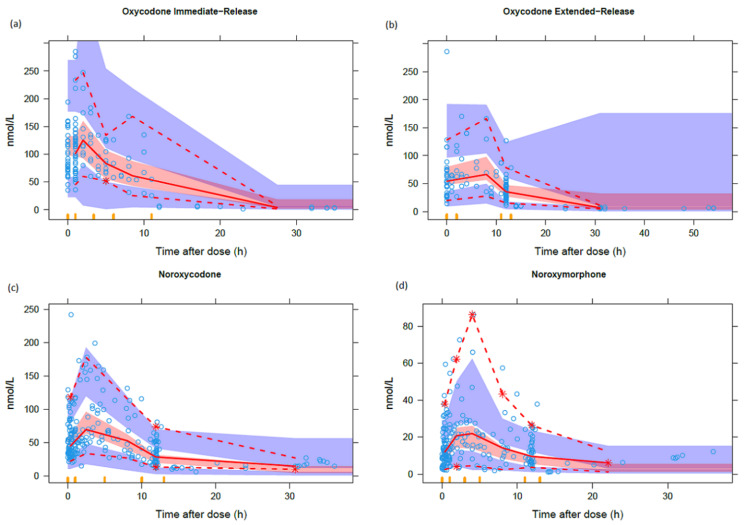
Prediction-corrected visual predictive checks of the final pharmacokinetic model of (**a**) oxycodone immediate-release, (**b**) oxycodone extended-release, (**c**) nor-oxycodone and (**d**) nor-oxymorphone (*n* = 1000). The solid red lines represent the median of the observed values. The dashed lines represent the 5 and 95% percentiles of the observed values. The red shaded areas represent the model-predicted median (with 95% confidence intervals for the prediction). The blue shaded areas represent the model-predicted 5 and 95% percentiles (both with 95% confidence intervals for the prediction).

All visual and numerical model evaluations showed that the model predicted the pharmacokinetics of oxycodone, nor-oxycodone and oxymorphone well. The visual predictive checks are shown in Figure 3. Other visual diagnostic evaluations are shown in Figures S1–S4.

### 3.3. Pharmacodynamic Analysis

Five patients were excluded for the pharmacodynamic analysis due to concomitant fentanyl use. A joint PK/PD NONMEM model was developed as specified in the methods. However, because patient-reported pain did not inversely follow oxycodone exposure, this was not feasible. 

The results of the mixed-effect models are depicted in Table 3. We found no association between oxycodone exposure and average pain (β 1.28, 95% CI −3.71–6.29) or maximal pain (β 0.58, 95% CI −5.74–6.89). Further, we found no association between oxycodone exposure and adverse events (β −2.72, 95% CI −8.57–3.12), no association between nor-oxycodone exposure and adverse events (β −3.29, 95% CI −10.62–4.04), and no association between nor-oxymorphone exposure and adverse events (β −12.13, 95% CI −39.43–15.18).

## 4. Discussion

We developed a model to describe the pharmacokinetics of oxycodone including the metabolites, nor-oxycodone, and nor-oxymorphone in patients with cancer-related pain. The similarities between reported values in the literature, and the model diagnostics, confirm the adequacy of the model. All compounds were sufficiently described by a one-compartment model for oxycodone nor-oxycodone and nor-oxymorphone, with the first-order absorption of IR and ER oxycodone, the first-order formation of metabolites and first-order elimination. The absorption rates from both IR and ER tablets were similar to those described in prior research [12,17]. Multiple models have been used to describe the absorption pattern from ER tablets, including a first-order process [11], with and without lag times [13,17,30] and Weibull absorption [12]. The consensus, however, is that the absorption from ER formulations is biphasic, as stated in the summary of product characteristics [31]. However, our data did not support biphasic absorption, most likely because most samples were taken 12 h after ER oxycodone. Denser sampling during the absorption phase is required to model the biphasic release from oxycodone ER tablets [30]. Oxycodone clearance and inter-individual variability on this parameter were similar to previous literature and the specifications of the manufacturer [8,13,17,31]. This indicates that the pharmacokinetics of oxycodone in cancer patients are similar to patients without comorbidities.

After introduction of the covariates, none significantly improved the pharmacokinetic model. In part, this could be attributed to the relatively low number of patients, no Child-Pugh class B or C and the narrow distribution of parameters for age and eGFR [5,8,17,32]. The UGT2B7 enzyme is involved in oxycodone glucuronidation, and we found a non-significant trend towards decreased oxycodone metabolism when a patient harbored a T allele in the UGT2B7 gene. No significant difference in the conversion rate from nor-oxycodone to nor-oxymorphone was seen between CYP2D6 extensive and intermediate metabolizers. However, because we did not model oxymorphone and, therefore, did not quantify every route to nor-oxymorphone conversion, we could not exclude the influence of the CYP2D6 genotype. In addition, we found no difference between patients harboring wild-type and heterozygous CYP3A4*22 genotypes in the conversion rate of oxycodone to nor-oxycodone in this model. Although prior research did prove the influence off strong inhibitors on the formation of nor-oxycodone [33], the role of the CYP3A4*22 genotype has not (yet) been proven [34]. 

Whereas nor-oxymorphone and nor-oxycodone poorly cross the blood–brain barrier, the analgesic effect of oxymorphone remains the subject of discussion [13,35,36,37]. Despite low plasma concentrations, which are approximately 2% that of oxycodone, and a relatively low presence in cerebrospinal fluid, oxymorphone is believed to contribute 15–20% of the total analgesic effect after oral administration [8,9,37,38,39]. This is mainly because the receptor affinity of oxymorphone is 8–44 times higher than oxycodone. As more than half of the oxymorphone samples were BLQ in this study, excluding these would introduce a significant bias in the model [40]. In order to fully elucidate the role of oxymorphone in analgesia, a more sensitive quantification method should be developed.

Some factors affected the association between oxycodone, nor-oxycodone, and nor-oxymorphone exposure with pain scores and adverse events. Although patients were asked to rate the average and maximal pain for the previous 12 h, the 12 h time-interval leaves space for external factors to influence patients’ pain perception and retrieval. Furthermore, although all patients had nociceptive pain, some also experienced neuropathic pain. Eight out of 28 patients used amitriptyline, gabapentin, or pregabalin, all of which are agents used to treat neuropathic pain, which could have affected the results. A more timely collection of pain scores could help extract oxycodone and oxycodone metabolite analgesic action from overall pain scores. 

In the introduction, we hypothesized that oxycodone metabolites could contribute to the occurrence and severity of adverse events. In our model, the AUC of nor-oxymorphone was eight times lower than the AUC of oxycodone. The AUC of nor-oxycodone was two thirds that of oxycodone. Combined with their potency to inhibit the μ-opioid receptor, the metabolites could contribute 40% of all peripheral adverse events. However, in this study, we could not find an association between the occurrence and severity of adverse events and oxycodone and oxycodone metabolite exposure. This is mainly due to limitations of this study, such as a small sample size. In this study (EMC 09-332), patients that were treated clinically with opioids for moderate-severe pain were included, and in this setting, oxycodone is used less frequently than other opioids as oxycodone is not available for parenteral titration in our hospital. Furthermore, our results could have been influenced by the prophylactic and as needed treatment of adverse events. Prophylactic treatment with laxatives is initiated at the start of oxycodone treatment to prevent constipation when opioids are prescribed. Additionally, anti-emetics are also prescribed as needed at the start of oxycodone treatment. Accordingly, some gastrointestinal adverse events could therefore be missed or underestimated, which may have affected the outcomes. Similar to pain scores, a more timely collection of adverse events in a larger cohort, could help to determine to what extent oxycodone metabolites contribute to the occurrence and severity of adverse events.

Achieving adequate pain control remains a challenge in patients with cancer-related pain [1,41]. To help optimize treatment for these patients, modelling can help to unravel the relationship between PK/PD for oxycodone and its metabolites. In addition to prior research on this topic, we developed a model that describes oxycodone metabolite pharmacokinetics and associated this with pharmacodynamic endpoints. To effectively associate oxycodone metabolite exposure with clinically relevant endpoints, real-world data from a large population and dense collection of pain-scores and adverse events after administration are essential. With additional data, this model could be used to associate oxycodone metabolite exposure with pharmacodynamic endpoints. In addition, denser plasma-sampling following oxycodone ER administration is needed to accurately describe the biphasic absorption from ER oxycodone tablets. Furthermore, a more sensitive method of oxymorphone quantification needs to be developed. More oxymorphone measurements could stabilize a complete pharmacokinetic model incorporating oxycodone and its metabolites.

## 5. Conclusions

In conclusion, we developed the first model to describe the pharmacokinetics of oxycodone, including the metabolites nor-oxycodone, and nor-oxymorphone in hospitalized patients with cancer pain. No covariates were found that influence oxycodone pharmacokinetics. Moreover, no association between oxycodone pharmacokinetics and pharmacodynamics was found. Additional research, including more patients and a more timely collection of PD data, is needed to further elucidate oxycodone and oxycodone metabolite PK/PD relationships. This model is an important starting point for further studies to optimize oxycodone dosing regiments in patients with cancer-related pain.

## Figures and Tables

**Table 1:** Patient characteristics.

Patient Characteristics (*n* = 28)	No./Median	%/Range
Sex (female)	12	43%
Age (years)	62.5	39–81
Weight (kg)	80.0	46–135
Body Mass Index	27.7	18.9–42.6
Follow-up (hours)	80.7	24.1–211.4
No. of samples/patient	12	4–51
Dose at inclusion*		
Immediate release (mg)	5	0–25
Extended release (mg)	10	0–40
Primary tumor		
Urogenital	9	32%
Breast	5	18%
GIST/Soft tissue sarcoma	4	14%
Melanoma	3	11%
Other	7	25%
WHO performance status		
0	0	0%
1	8	29%
2	9	32%
3	4	14%
Unknown	7	25%
eGFR (mL/min)	86.0	37–>90
Albumin (g/L)	42.5	29–49
CYP2D6		
EM	12	43%
IM	10	36%
Missing	6	21%
CYP3A4*22		
*1/*1	22	79%
*1/*22	6	21%
*22/*22	0	0%
UGT2B7*2		
Wildtype	6	21%
Heterozygous	17	61%
Variant	5	18%
Pain scores at inclusion		
Average pain	2	0–7
Maximal pain	6	0–10

* Five patients did not use immediate-release oxycodone formulations during the study. Four patients did not use extended-release oxycodone formulations during the study.

**Table 2:** Parameter estimations and bootstrap results of the joint oxycodone, nor-oxycodone and nor-oxymorphone model.

Parameter (Unit)	Parameter Estimate [Shrinkage]	RSE (%)	Bootstrap Median	95% CI Bootstrap
K_a, IR_ (h^−1^)	3.61	39.3	3.33	(0.13–6.22)
K_a, ER_23 (h^−1^)	0.329	38.6	0.311	(0.11–3.14)
V3/F (L)	619	9.2	600	(205–771)
K30 (h^−1^)	0.012	FIX	0.012	FIX
K34 (h^−1^)	0.086	10.9	0.087	(0.07–0.27)
V4/F (L)	16.3	FIX	16.3	FIX
K40 (h^−1^)	3.28	30.1	3.22	(1.53–4.99)
K45 (h^−1^)	1.36	32.1	1.47	(0.33–2.16)
V5/F (L)	64.1	FIX	64.1	FIX
K50 (h^−1^)	1.97	35.9	2.00	(0.47–3.59)
**IIV**				
K34 (CV%)	35.7 [5.4]	14.8	34.8	(22.3–46.8)
K40 (CV%)	98.7 [3.4]	26.0	96.7	(52.3–241)
K50 (CV%)	81.7 [7.1]	10.8	78.4	(55.6–102)
**Residual error**				
Oxycodone	[8.6]			
**Proportional (%)**	39.7	13.4	38.7	(28.8–51.3)
**Additive (nM)**	0	FIX	0	FIX
Nor-oxycodone	[5.4]			
**Proportional (%)**	16.7	18.5	16.5	(9.78–23.3)
**Additive (nM)**	3.34	40.4	3.10	(0.77–6.34)
Nor-oxymorphone	[4.0]			
**Proportional (%)**	15.6	21.5	15.9	(7.57–22.7)
**Additive (nM)**	1.09	24.3	0.98	(0.25–1.65)
**Conditional number**	517.28			

V3: Central distribution compartment of oxycodone, V4: Central distribution compartment of nor-oxycodone, V5: Central distribution compartment of nor-oxymorphone, IIV: Inter-individual variability. K_a,IR_ and K_a,ER_ are the absorption rates for IR and ER tablets. K30, K40 and K50 are the elimination rate constants for oxycodone, nor-oxycodone and nor-oxymorphone.

**Table 3:** Results of the mixed-effects linear regression regarding exposure–response associations ^a^.

Observations	Pain		Adverse Events
	Average	Maximal	Sum
Oxycodone exposure (mM/hour)	1.28 (−3.73–6.29) ^b^	0.58 (−5.74–6.89) ^b^	−2.72 (−8.57–3.12) ^b^
Nor-oxycodone exposure (mM/hour)	NA	NA	−3.29 (−10.62–4.04) ^b^
Nor-oxymorphone exposure (mM/hour)	NA	NA	−12.13 (−39.43–15.18) ^b^
Number of observations	100	125	110
R^2^	0.478	0.524	0.251

^a^ Results are reported as ‘β (95% confidence interval)’ ^b^ *p* > 0.05.

## Data Availability

The datasets and code generated during and/or analyzed during the current study are available on reasonable request.

## Supplementary Materials

The following are available online at https://www.mdpi.com/article/10.3390/cancers13112768/s1, Figure S1–S3: Goodness-of-fit plots for oxycodone, nor-oxycodone and nor-oxymorphone, Figure S4: Normalized Prediction Distribution Error plots, Table S1: CYP3A4 and CYP2D6 inducers and inhibitors, Document S1: Covariate equations, Document S2: Control stream of the final model.
