# Supplementary material for: Population Pharmacokinetics of Oxycodone and Metabolites in Patients with Cancer-Related Pain
Source: Cancers (Basel). 2021 Jun 2;13(11):2768. doi: 10.3390/cancers13112768 (PMC8199682; doi:10.3390/cancers13112768)
Supplement: Supplementary file 1 [file cancers-13-02768-s001.zip › cancers-1211335-supplementary.pdf]

Appendix A

Table S1: List of intermediate and strong inducers and inhibitors of CYP3A4 and CYP2D6. Compounds in bold are strong inhibitors/inducers. Based on the KNMP kennisbank from the Royal Dutch Pharmacists Associations and the FDA Drug Development and Drug Interaction: Table of Substrates, Inhibitors and Inducers.

**CYP3A4**		**CYP2D6**	
Inhibitors	*Inducers*	*Inhibitors*	*Inducers*
**Clarithromycin**	**Carbamazepine**	**Bupropion**	None
**Cobicistat**	**Efavirenz**	**Cinacalcet**	
**Erythromycin**	**Enzalutamide**	**Fluoxetine**	
**Itraconazole**	**Hypericum**	**Quinidine**	
**Ketoconazole**	**Mitotane**	**Paroxetine**	
**Posaconazole**	**Nevirapine**	**Terbinafine**	
**Ritonavir**	**Phenobarbital**	Abiraterone	
**Voriconazole**	**Phenytoin**	Duloxetine	
Aprepitant	**Primidone**	Mirabegron	
Crizotinib	**Rifabutin**		
Diltiazem	**Rifampicin**		
Fluconazole	Apalutamide		
Fluvoxamine	Bosentan		
Imatinib			
Verapamil			

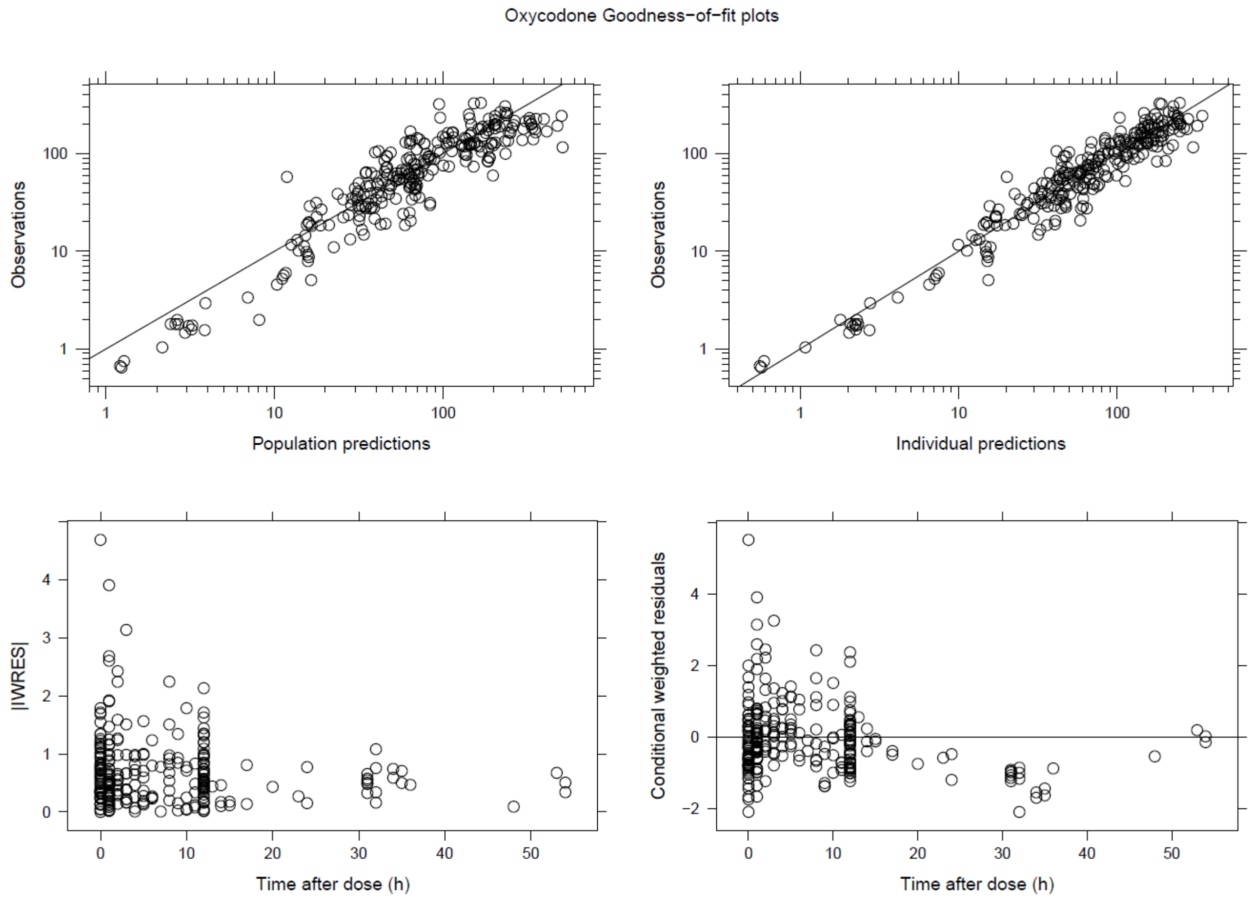

Figure S1: Goodness of fit plots (GOF) of oxycodone. Top left: log-transformed population predictions vs. observations with a unity line. Top right: Log transformed individual predictions versus observations with a unity line. Bottom left: Individual weighed residual errors (IWRES) vs. Time after dose. Bottom right: Conditional weighted residual errors vs. Time after dose.

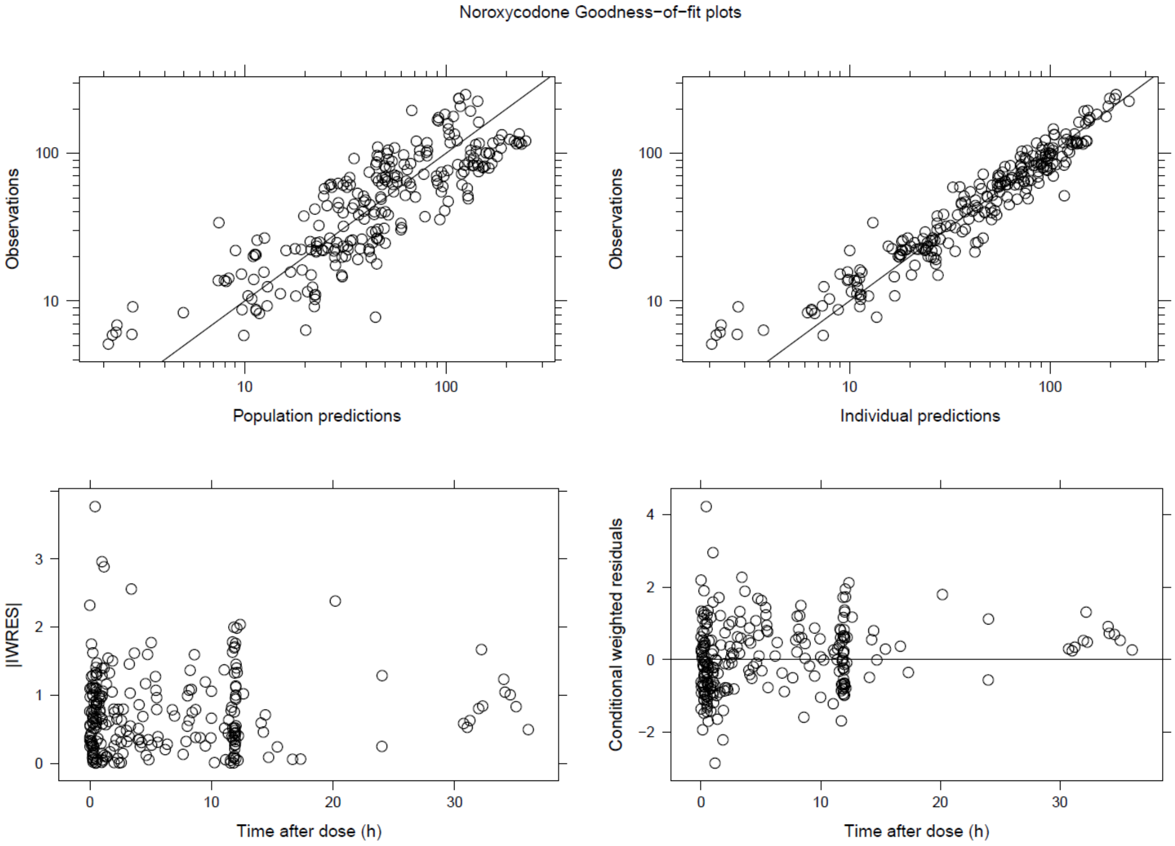

Figure S2: Goodness of fit plots (GOF) of nor-oxycodone. Top left: log-transformed population predictions vs. observations with a unity line. Top right: Log transformed individual predictions versus observations with a unity line. Bottom left: Individual weighed residual errors (IWRES) vs. Time after dose. Bottom right: Conditional weighted residual errors vs. Time after dose.

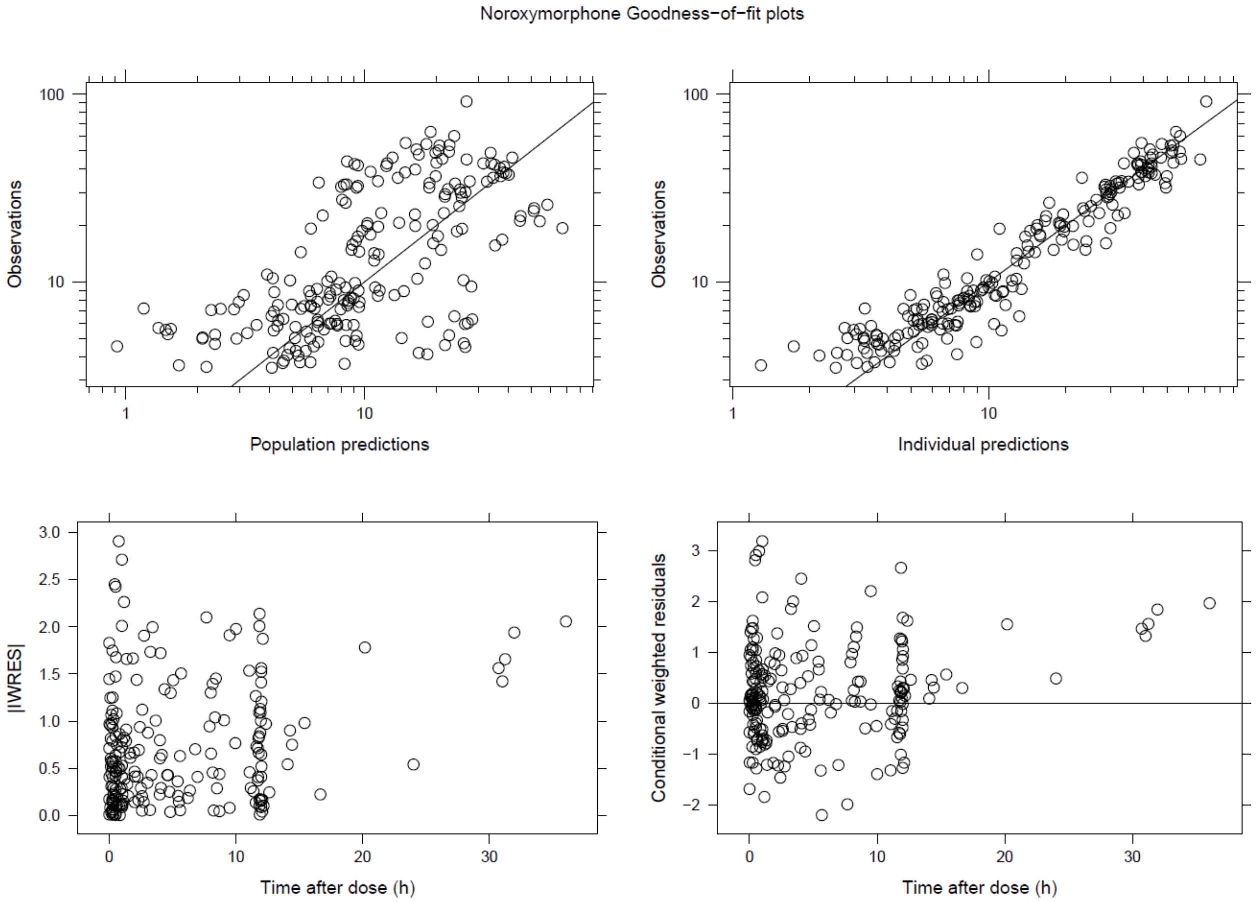

Figure S3: Goodness of fit plots (GOF) of nor-oxymorphone. Top left: log-transformed population predictions vs. observations with a unity line. Top right: Log transformed individual predictions versus observations with a unity line. Bottom left: Individual weighed residual errors (IWRES) vs. Time after dose. Bottom right: Conditional weighted residual errors vs. Time after dose.

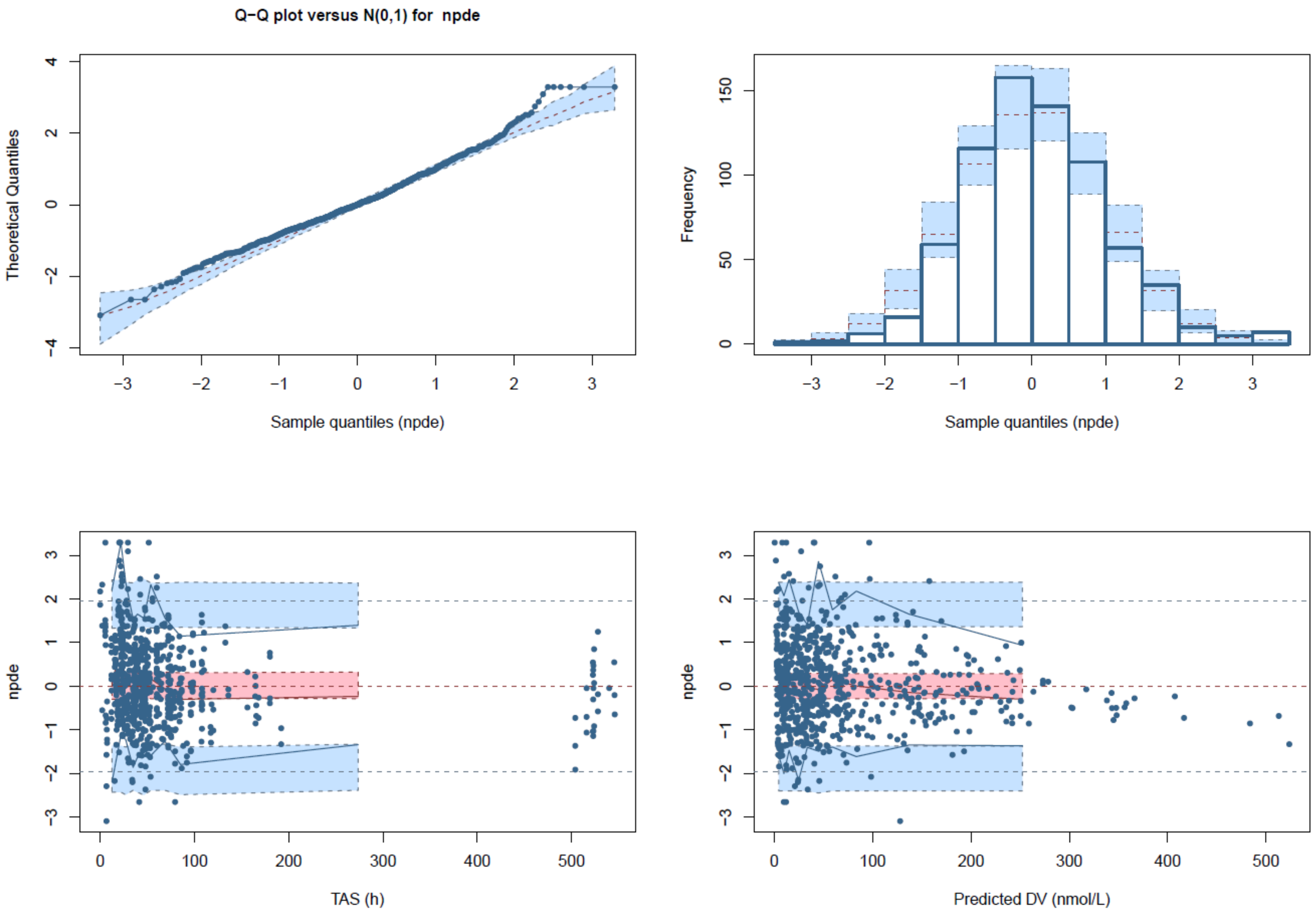

Figure S4: Normalized Prediction Distribution Error (NDPE) plots. Top left QQ plot between observed and simulated data. Top right: Histogram of NPDE. Light blue areas: theoretical normal distribution. Bottom left: Scatterplot of NPDE versus time after start (TAS). Bottom right: Scatterplot of NPDE versus predicted plasma concentrations (DV).

Document S1: Covariate equations

Covariate equations:

Power model:

$$P_i = CL_{pop} * \left(\frac{COV}{median\ COV}\right)^{\Theta_{COV}} * exp^{(\eta_i)}$$

Exponential model:

$$P_i = CL_{pop} * \left(\frac{COV}{median\ COV}\right) * \Theta_{COV} * exp^{(\eta_i)}$$

Proportional model

$$P_i = CL_{pop} * \ {\Theta_{Cat}}^{FLAG} * exp^{(\eta_i)}$$

Genotype models

Dominant model

$$P_i = CL_{pop} * \ {\Theta_{GEN}}^{FLAG(VAR+HZ)} * exp^{(\eta_i)}$$

Recessive model

$$P_i = CL_{pop} * \ {\Theta_{GEN}}^{FLAG(VAR)} * exp^{(\eta_i)}$$

Exponential model

$$P_i = CL_{pop} * ((\Theta_{GEN} * HZ) + (2 * \Theta_{GEN} * VAR)) * exp^{(\eta_i)}$$

Document S2: Control stream of the final model:

FINAL MODEL CONTROL STREAM

```
$PROBLEM PK
$INPUT CENSOR ID TIME AMT DV EVID MDV TAD CMT HT WT AGE IR GNDR TYPE BMI ALBU
CREAT CYP2D6 CYP3A4 UGT2B7
$DATA PKPD_Oxy_181220COV.csv IGNORE=C

$SUBROUTINES ADVAN6 TOL=3

$MODEL
	NCOMPS=5
	COMP (ABSIR, DEFDOSE)		;DOSE COMPARTMENT IR
	COMP (ABSER)		;DOSE COMPARTMENT ER
	COMP (CENTRAL)		;CENTRAL COMPARTMENT
	COMP (METAB1)		;NOROXYCODONE
	COMP (METAB3)		;NOROXYMORPHONE

$PK
TVV3 = THETA(4)
V3 = TVV3

TVV4 = THETA(7)
V4 = TVV4

TVV5 = THETA(12)
V5 = TVV5

;scaling from nmol/L (observations) to mmol (dosages)
S3 = V3/1000000
S4 = V4/1000000
S5 = V5/1000000

;when a patient was sampled prior to the first dosage in the study
 IF (A_0FLG.EQ.1) THEN
	IF (MDV.EQ.0) THEN
	A_0(3) = DV*S3
	A_0(4) = NOROXY*S4
	A_0(5) = NORMOR*S5
	ENDIF
 ENDIF

 K13 = THETA(5)
 K23 = THETA(6)
 K30 = THETA(3)
 K34 = THETA(9) * EXP(ETA(1))
 K40 = THETA(8) * EXP(ETA(2))
```

```
 K45 = THETA(14)
 K50 = THETA(13) * EXP(ETA(3))


$THETA
(0, 0.343)              ;1 PROP ERR OXYCODONE
(0) FIX                 ;2 ADD ERR OXYCODONE
(0, 0.01224) FIX        ;3 K30
(0, 566)                ;4 V3
(0, 3.52)         ;5 K13
(0, 0.385)              ;6 K23
(0, 16.3) FIX           ;7 V4
(0, 3.256)              ;8 K40
(0, 0.0888)             ;9 K34
(0, 0.177)              ;10 PROP ERR NOROXYCODONE
(0, 3.29)               ;11 ADD ERR NOROXYCODONE
(0, 64.1) FIX           ;12 V5
(0, 1.975)              ;13 K50
(0, 1.51)         ;14 K45
(0, 0.177)              ;15 PROP ERR NOROXYMORPHONE
(0, 1.29)         ;16 ADD ERR NOROXYMORPHONE

$DES
DADT(1)= -K13*A(1)
DADT(2)= -K23*A(2)
DADT(3)= K13*A(1) + K23*A(2) - K30*A(3) - K34*A(3)
DADT(4)= K34*A(3) - K40*A(4) - K45*A(4)
DADT(5) = K45*A(4) -K50*A(5)

$ERROR                          ;; M3 method CMT specific
 IPRED = F
 IRES = DV-IPRED

 IF(CMT.EQ.3) W1 = IPRED*THETA(1)+THETA(2)
 IF(W1.EQ.0) W1 = 1
 IF(CMT.EQ.3.AND.TYPE.EQ.1) THEN
        IWRES = IRES/W1
        Y = IPRED+W1*ERR(1)
        F_FLAG = 0
 ENDIF

 IF(CMT.EQ.4) W2 = IPRED*THETA(10)+THETA(11)
 IF (W2.EQ.0) W2 = 1
 IF(CMT.EQ.4.AND.TYPE.EQ.1) THEN
        IWRES = IRES/W2
        Y = IPRED+W2*ERR(2)
        F_FLAG = 0
 ENDIF
```

```
 IF(CMT.EQ.5) W3 = IPRED*THETA(15)+THETA(16)
 IF (W3.EQ.0) W3 = 1
 IF(CMT.EQ.5.AND.TYPE.EQ.1) THEN
             IWRES = IRES/W3
             Y = IPRED+W3*ERR(3)
             F_FLAG = 0
 ENDIF

 IF(CMT.EQ.3)LLOQ = 0.6342
 IF(CMT.EQ.4)LLOQ = 3.318511
 IF(CMT.EQ.5)LLOQ = 3.480561

 IF(CMT.EQ.3.AND.TYPE.EQ.2) THEN
  F_FLAG = 1
  DEL=0.000001
  DUM = (LLOQ - IPRED)/(W1+DEL)
  CUMD = PHI(DUM)
  Y = CUMD
  MDVRES = 1
 ENDIF

 IF(CMT.EQ.4.AND.TYPE.EQ.2) THEN
  F_FLAG = 1
  DEL=0.000001
  DUM = (LLOQ - IPRED)/(W2+DEL)
  CUMD = PHI(DUM)
  Y = CUMD
  MDVRES = 1
 ENDIF

 IF(CMT.EQ.5.AND.TYPE.EQ.2) THEN
  F_FLAG = 1
  DEL=0.000001
  DUM = (LLOQ - IPRED)/(W3+DEL)
  CUMD = PHI(DUM)
  Y = CUMD
  MDVRES = 1
 ENDIF

$OMEGA BLOCK(3)
 0.134 ; K34
 0.107 0.277 ;K40
 0.01 0.01 0.03 ;K50

$SIGMA
 1 FIX ;OXYCODON
 1 FIX ;NOROXYCODON
```

```
 1 FIX ;NOROXYMORFON

$EST METHOD=COND INTER NOABORT MAXEVAL=9000 NOABORT LAPLACIAN SIG=2 PRINT=1
POSTHOC
$COV PRINT=E
```
